# Effectiveness of Physiotherapy for Improving Participation, Gross Motor Function, Gait and Balance in Children and Adolescents with Cerebral Palsy: Study Protocol for a Randomized Controlled Trial

**DOI:** 10.3390/jcm14072214

**Published:** 2025-03-24

**Authors:** Soraya Pacheco-da-Costa, Isabel Rodríguez-Costa, Vanesa Abuín-Porras, Ángel Asúnsolo-del-Barco, Victoria Calvo-Fuente, Concepción Soto-Vidal

**Affiliations:** 1 Neuromusculoskeletal Physical Therapy in Stages of Life Research Group (FINEMEV), Department of Nursing and Physical Therapy, Faculty of Medicine and Health Sciences, Universidad de Alcalá, Autovía A2, km 33,200, 28805 Alcalá de Henares, Spain; soraya.pacheco@uah.es (S.P.-d.-C.); conchi.soto@uah.es (C.S.-V.); 2 Humanization in the Intervention of Physiotherapy for the Integral Attention to the People (HIPATIA), Department of Nursing and Physical Therapy, Faculty of Medicine and Health Sciences, Universidad de Alcalá, Autovía A2, km 33,200, 28805 Alcalá de Henares, Spain; 3 Department of Physiotherapy, Faculty of Medicine, Health and Sports, European University of Madrid, 28670 Villaviciosa de Odón, Spain; 4 Public Health and Epidemiology Research Group (ISPE), Medicine Degree, Department of Surgery, Social and Medical Sciences, Faculty of Medicine and Health Sciences, Universidad de Alcalá, Autovía A2, km 33,200, 28805 Alcalá de Henares, Spain; angel.asunsolo@uah.es

**Keywords:** cerebral palsy, physical therapy, clinical trial, functional goals, task oriented, task-specific training, participation, gross motor function, gait, balance

## Abstract

**Background:** People with cerebral palsy (CP) present with limitations in gait and functionality, with a great impact on participation. Physiotherapy interventions based on goal-directed training (GDT), treadmill gait training (TGT), and action observation treatment (AOT) showed to be effective for improving functionality, gait and balance in children and adolescents with CP. On the other hand, since COVID-19 lockdown, telecare has an increased role in physiotherapy interventions. The aim of this randomized controlled trial (RTC) is to analyze the effectiveness of a multimodal intervention that combines face-to-face PT sessions of GDT and TGT and online PT sessions of a family-centered education program, which includes AOT activities and is effective to improve participation, gross motor function, gait and balance in children and adolescents with CP. **Methods:** A single-blinded RCT is proposed for 48 children and adolescents with CP (6–17 years old) who will be randomly allocated into two groups: the experimental group will receive six weeks of a multimodal PT intervention with 12 face-to-face sessions (GDT and TGT) and 6 online sessions of a family-centered telecare EP, which includes AOT activities. Participants in the control group will carry on with their regular PT treatment plus the EP as the experimental group. The outcome variables of participation (CAPE); gait speed and endurance (10 mm/6 mm); gross motor function (GMFM-88-SP); and dynamic and static balance (PBS) will be collected at baseline, after group interventions and 12 weeks from baseline and will be compared following standard principles for RCTs. **Conclusions**: The implementation of a multimodal PT intervention that combines face-to-face sessions of GDT and TGT and online sessions of a family-centered EP, which includes AOT activities, may be effective to improve participation, gross motor function, gait and balance in children and adolescents with CP. Trial registration: ClinicalTrials.gov with ID: NCT04778930.

## 1. Introduction

Cerebral palsy (CP) is **a** non-progressive neurological disorder caused by damage to the developing brain during the pre, peri, or postnatal period [1,2,3]. It is the most common cause of motor disability in childhood, affecting movement, posture, and coordination. The overall birth prevalence of CP is estimated to be 1.6 per 1000 live births worldwide [4]. Despite its non-progressive nature, CP leads to chronic impairments that can evolve over time, affecting an individual’s functional independence and participation in different settings, such as family, school and community.

CP children and adolescents might have a broad spectrum of impairments in body structure and function, including muscle tone alteration, postural instability, motor coordination deficits, and impaired voluntary movement control. These impairments can significantly impact functional activities and restrict participation in essential aspects of daily life, such as family interactions, education, social activities, and community engagement [5,6,7].

Children and adolescents with CP commonly experience limitations in independent mobility, primarily due to deficits in gross motor function, gait, and balance. These mobility challenges significantly affect their ability to explore the environment, engage in play, and perform different activities of daily living. Consequently, restricted mobility and participation may lead to secondary complications, including muscle contractures, joint deformities, and reduced cardiovascular fitness, which further exacerbate functional limitations [7,8,9].

Given these challenges, physiotherapy (PT) interventions play a crucial role in managing individuals with CP by addressing impairments, enhancing functional abilities, and promoting participation in meaningful activities [10,11]. Effective PT programs should adopt a holistic and individualized approach, focusing not only on improving body structure and function but also on fostering independent mobility, participation, and overall well-being.

Recent advances in research have emphasized evidence-based PT approaches that enhance mobility, function, and participation in children and adolescents with CP. Systematic reviews [12,13,14] and international clinical practice guidelines for CP interventions [15] support the implementation of task-oriented and motor learning-based interventions. Among them, goal-directed training (GDT), also referred as task-specific training (TST), is an approach that focuses on practicing meaningful, goal-oriented tasks that directly translate into improved daily function [16,17]; treadmill gait training (TGT) is widely recognized as an effective intervention for improving gait parameters, endurance, and balance in children with CP and can enhance walking ability, step symmetry, and lower limb strength, promoting independent ambulation [18,19,20]; and research indicates that action observation therapy (AOT) can significantly improve motor function, coordination, and movement efficiency [21,22,23]. AOT is an emerging motor learning-based intervention that uses mirror neuron system to enhance motor planning and execution. In AOT, children and adolescents with CP observe goal-directed actions performed by their peers, either through video demonstrations or live models, and then attempt to replicate the movements [21,22,23]. All the previously mentioned interventions work best when they are family-centered, have regular attention, follow-up and feedback by therapists, and include education programs to empower children, adolescents and their families in the achievement of functional goals [21,22,24]. The intervention goals and activities should be established among children and adolescents with CP and their families and caregivers, supported by therapists [15].

Due to the health situation caused by COVID-19, health services had to adapt, and a new vision through telecare, also called telerehabilitation, was necessary to provide alternatives for PT interventions to children and adolescents with CP [25,26]. Telecare involves the remote delivery of PT services via digital platforms, allowing real-time therapist supervision, exercise guidance, and caregiver education. PT interventions play an important role in the maintenance of function and the follow-up of individuals, in addition to giving the opportunity for Physiotherapists to be in the natural environment of the child and adolescent [27,28].

Therefore, the aim of this randomized controlled trial (RTC) is to analyze the effectiveness of a multimodal PT intervention that combines face-to-face sessions of GDT and TGT and online sessions of a family-centered EP, which includes AOT activities, to improve participation, gross motor function, gait and balance in children and adolescents with CP.

## 2. Materials and Methods

The study protocol follows the Standard Protocol Items: Recommendations for International Trials (SPIRIT) [29].

### 2.1. Study Design

A single-blinded randomized controlled trial will be conducted according to Consolidated Standards of Reporting Trials (CONSORT) guidelines [30,31].

### 2.2. Ethics Approval and Consent to Participate

This study complies with the ethical principles of the Declaration of Helsinki [32] and the Spanish Law on Personal Data Protection and Guarantee of Digital Rights [33]. The RTC Efectividad de la Fisioterapia para la mejora de la funcionalidad de la marcha y participación en el entorno en niños y adolescentes con Parálisis Cerebral: ensayo clínico aleatorio (FIMAPACE) was approved by the Ethics Committee of Animal Experimentation and Research of the University of Alcalá with the code CEI/HU/2020/39, and it is registered in the www.clinicaltrials.gov (accessed on 7 February 2023) database under number NCT04778930. Written and informed consent will be obtained from parents or guardians, and all participants will give their assent upon entering the trial, before matching and randomization. Information sessions about this study will be held for parents/guardians and participants, where an information sheet will be provided.

Once informed, parents/guardians will read and sign the informed consent. All the information collected from each of the participants will be filed in a personal PT individual file, to which a file number will be assigned in chronological order of data collection, where data collected on paper and computer data extracted from the various tests will be entered.

### 2.3. Sample Size Calculation

The authors have not found meta-analyses nor systematic reviews about the impact of the PT interventions for improving participation. The meta-analyses and systematic reviews consulted on different interventions for improving the functionality of children and adolescents with CP include samples from 10 to 30 participants [12,16,18,34,35,36]. Study samples from the CP population are not very large due to the variety of interventions and parameters for assessing function in PT interventions. As far as the authors know, there is no consensus on the parameter or predicted change in order to better estimate a sample size. Therefore, in this study, authors opted to obtain a larger sample than most previously published studies, considering 40 participants (20 in each group) for inclusion. Considering a 20% dropout rate, a total of 48 participants should be included—24 in the experimental group (EG) and 24 in the control group (CG). At the end of this study, the statistical power will be calculated.

### 2.4. Study Sample

Children and adolescents aged from 6 to 17 years old and diagnosed with CP will be recruited across schools in Madrid and Guadalajara, Spain. The estimated population for potential participants in this RCT is 150 people, who will be identified through their school Physiotherapists.

- Inclusion criteria: children and adolescents with CP, aged between 6 and 17 years old; levels I-III of Gross Motor Function Classification (GMFCS) [37]; Manual Abilities Classification System (MACS) [38]; and Communication Function Classification System (CFCS) [39].
- Exclusion criteria: children and adolescents who have received a botulinum toxin injection in their lower limbs 6 weeks before this study or during the intervention; have undergone surgery on lower limbs in the 6 months prior to their participation in this study or during this study; or who present inability to follow the planned program due to moving to another city or another reason.

### 2.5. Randomization and Blinding

A total of 48 participants will be randomly allocated in 2 groups, with 24 participants in each group. The sequence will be generated randomly by computer and the participants will be allocated to EG or CG.

Two members of the research team will perform the assessments and will be blinded to group and treatment assignments.

### 2.6. Study Flow

**Figure 1:**
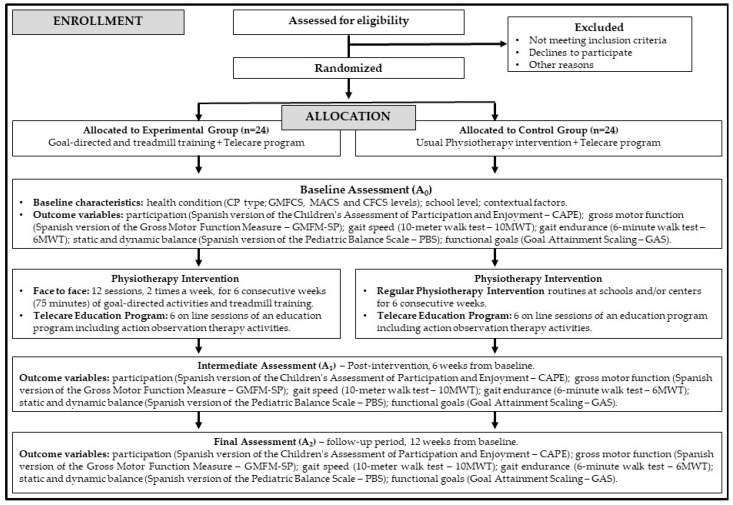
Flow chart of this study. Abbreviations: CP, cerebral palsy; GMFCS, Gross Motor Function Classification System; MACS, Manual Abilities Classification System; CFCS, Communication Function Classification System.

Figure 1 shows the flow chart of this RCT following the CONSORT guidelines [30,31].

**Figure 2:**
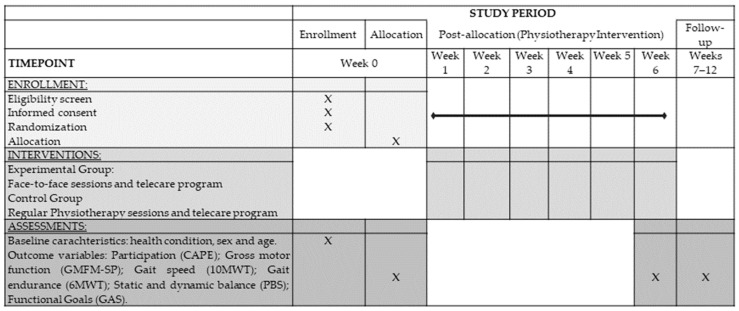
SPIRIT schedule of enrollment, interventions, and assessments [29].

Figure 2 shows the schedule of enrollment, interventions, and assessments based on the SPIRIT statement [29].

### 2.7. Outcome Variables

After randomization, sociodemographic, clinical, and outcome variables will be collected at baseline assessment (A_0_). PT interventions will be carried out in each group for 6 weeks. After group interventions, an intermediate assessment (A_1_) will be performed, and a follow-up assessment (A_2_) will be carried out 12 weeks from baseline (Table 1). The assessments will be carried out by 2 Pediatric Physiotherapists (PPT), members of the research team, after training and consensus meetings.

Primary outcomes are as follows:

Participation: measured with the Spanish version of the Children’s Assessment of Participation and Enjoyment—CAPE, which has good internal consistency, construct validity, convergent and discriminant [40]. This instrument measures participation in extracurricular leisure activities in children and adolescents with and without disabilities during the last 4 months. It includes 55 activities, and each activity is categorized by domains (Formal, Informal) and type (Recreational, Physical, Social, Skills-based and Self-Improvement). In addition, it also measures the diversity and intensity of participation, with whom they participate and where, and the enjoyment in performing the activity.

Gross motor function: measured with the Spanish version of Gross Motor Function Measure—GMFM-SP [41], which consists of 88 items to assess the gross motor function in 5 dimensions: lying and rolling (A), sitting (B), crawling and kneeling (C), standing (D) and walking, running and jumping (E). From each dimension, a score of 0 to 100% is obtained, where a greater percentage means a greater number of items achieved. For this study, dimensions D and E are established as the goal dimensions. The minimum clinically important difference in children and adolescents with levels II and III in GMFCS is 1.5% and 1.2%, respectively [42].

Secondary variables are as follows:

Gait speed: measured with the 10 m walk test—10 MWT [43] in meters/second, which allows quick and easy assessing of walking speed. It is a commonly used test, both in clinical settings and in research, and it is outstanding for a high degree of intra- and inter-observer reliability [44].

Gait endurance: assessed with the 6 min walk test—6 MWT [45] that measures the distance, in meters, that an individual is able to walk for 6 min. It stands out for being easily reproducible, highly standardized and sensitive to changes, and it is the most reliable scale for the assessment of gait endurance [44].

Static and dynamic balance: assessed with the Spanish version of Pediatric Balance Scale—PBS [46]. It consists of 14 items to be scored from 0 to 4, 0 being the inability to perform the task and 4, the ability to perform the task following the guidelines described in each item without difficulty. The result is obtained by adding the score, which can reach a maximum of 56 points. The minimum clinically important difference is an increase in score between 3.66 and 5.83 points [47].

Functional goals: goal-setting will follow SMART criteria, and a maximum of 3 functional goals will be identified and defined by each participant [48,49]. They will be rated with the Goal Attainment Scaling (GAS), a 6-point-scale for measuring significant and realistic functional goals, and demonstrated to be a feasible outcome measure [50].

### 2.8. Interventions

#### 2.8.1. Experimental Group Intervention

Participants in the EG will complete a total of 18 sessions over six consecutive weeks, attending three sessions per week. Each week, they will engage in two face-to-face sessions focused on GDT and TGT, as well as six online sessions as part of a family-centered EP that integrates AOT activities. All PT sessions will be led by two highly experienced PPTs from the research team, each with over 10 years of expertise in treating children and adolescents with CP. The PPT will ensure individualized guidance, monitor progress, and adapt interventions to meet the specific needs of each participant. In order to support family, children and adolescents’ organization, each participant will receive a detailed schedule outlining the session agendas, including dates, times, formats (in-person and online), and key activities for each session. This will help families plan ahead, ensure consistent participation, and maximize the benefits of the program.

Face-to-face sessions will be held at the PT Unit of the Neuromusculoskeletal Physical Therapy in Stages of Life Research Group (FINEMEV) at University of Alcalá. Each PT session will last approximately 75 min. PT intervention will consist of 50–60 min of GDT, focusing on activities to activate the trunk, pelvis, and lower limb muscles for 15 min, followed by 45 min of functional training aimed at achieving specific functional goals based on the individual interests and needs of each participant. The final 15 min of the session will be dedicated to TGT on a treadmill, which will progressively increase in duration and speed. During the PT sessions, key aspects such as postural alignment and stability, movement components, selective motor control, functional movement sequences, task recognition, and motivation to complete the activities will be closely monitored and integrated into the intervention plan.

The telecare program will be carried out by remote assistance with the Alcalá University online campus, through the BlackBoard Ultra™ digital platform (https://uah.blackboard.com/ultra/institution-page, accessed on 17 January 2024), with 6 online sessions, 1 per week. The sessions will consist of approximately 60 min of a family-centered EP, which includes AOT activities [51]. The inclusion of a telecare EP program aims to enhance family involvement, provide additional guidance, and reinforce therapeutic strategies in a home-based setting, complementing their standard PT routines.

Session 1: Concept and causes of CP, general program goals and guidelines.

Session 2: The family-centered model: discussing the importance of involving family in the development of goals and tasks included in the physical therapy intervention to translate the child and/or adolescent’s new learning to their natural environment and to promote collaboration with the treatment team’s guidelines. To offer opportunities to discover what they want to achieve, clarify what they need to achieve those goals, and recognize what they already know and what they can do. To define what they still need to learn and do to improve children’s functional activity and participation in their environment.

Sessions 3, 4 and 5: AOT activities through observation of actions performed by other individuals to facilitate the activation of the same neural structures involved in the execution of the observed actions. According to participants’ age and functional level, videos will be recorded with actions that are familiar to them and with activities that are related to walking (getting up from/sitting in a chair, walking down a corridor, etc.) in their natural environment.

Session 6: Program evaluation with different activities on participation, learning strategies and integration of AOT activities into daily life.

**Figure 3:**
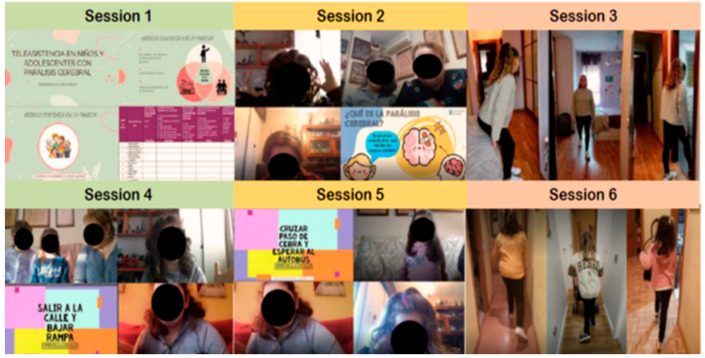
Some examples of the material used during the Telecare Program.

Figure 3 illustrates FIMAPACE telecare program.

#### 2.8.2. Control Group Intervention

Participants assigned to the CG will continue their regular PT intervention routines at their schools and centers for 6 weeks. In addition to that, they will also participate in the same family-centered EP as the EG. This ensures that all participants receive consistent educational support while maintaining their existing therapeutic interventions.

### 2.9. Statistical Analysis

First, a descriptive analysis of the study variables will be performed and, in the case of quantitative variables, the normality criteria will be checked. We will test whether there were differences after randomization between the two intervention groups. For this purpose, the appropriate bivariate tests will be used according to the type of variable. In a second stage, the effectiveness of the intervention will be analyzed, both in the short and medium term, adjusting for those variables that, according to previous analyses or due to the researchers’ consideration, could cause confusion. For these purposes, various logistic or linear regression models will be used (depending on the outcome variable). Finally, repeated measures models will be proposed in order to assess the evolution over time of the outcome variables.

The analysis will be performed both by protocol and by intention to treat.

The statistical package Statistical Package for the Social Sciences software (version 29, SPSS Inc., Chicago, IL, USA) or the statistical package STATA MP v18 (StataCop LLC, College Station, TX, USA) will be used.

## 3. Discussion

As far as the authors know, this study will be among the ones with a large sample-sized studies of PT interventions for children and adolescents with CP carried out so far.

Current intervention models for children and adolescents with CP are often costly, may have accessibility limitations, and frequently lack the necessary intensity to achieve optimal outcomes. Novak et al. [12] provide a comprehensive overview of the best available evidence for managing CP, emphasizing the effectiveness of interventions such as GDT, TGT, and AOT. Their review also highlights the role of medical and surgical treatments, including botulinum toxin injections and multilevel surgery. By systematically analyzing the literature, Novak et al. underscore the importance of a multidisciplinary approach to CP management, integrating various therapeutic strategies to maximize functional improvements and enhance overall outcomes.

In the proposed PT intervention for EG, key aspects such as establishing functional, integrating movement sequences and task recognition and motivation will be closely monitored. These elements will be actively integrated into the intervention plan to ensure a holistic approach tailored to the needs of each child or adolescent with CP. By addressing these factors, the intervention aims to enhance motor skills, improve functional independence, and foster greater engagement and motivation, ultimately promoting optimal outcomes in both short-term performance and long-term development.

In this context, telecare programs emerge as a critical component of comprehensive care for children and adolescents with CP. Telecare cannot fully replace in-person physiotherapy; it serves as a valuable complement to traditional rehabilitation models, offering flexible, accessible, and cost-effective solutions for long-term functional management. Future research should explore hybrid models that integrate in-person and remote PT interventions to optimize outcomes for children and adolescents with CP. Beckers et al. [52] discuss the significant potential of home-based training programs for PT interventions. They emphasize the adaptability of telecare services to meet individual needs and their role in overcoming geographical and accessibility barriers, thereby enhancing the equity of care access. This approach not only supports the continuity of care but also empowers families by integrating therapeutic exercises into daily routines, promoting greater adherence and engagement with the intervention programs. In addition, the inclusion of EP possibly facilitates adherence to treatment and the possibility of providing this approach to children and/or adolescents with different geographical locations and personal and/or social situations, contributing to equity of access. Besides, currently, AOT by telecare is increasingly being used in children and adolescents with CP, with favorable results [51,53].

Therefore, effective multimodal PT interventions that are personalized to align with individual functional goals and integrate active, meaningful task-specific training—enhanced by telecare—hold significant potential for improving motor abilities and promoting greater participation. By emphasizing task-oriented training in real-life contexts, these interventions facilitate the transfer of improved gait functionality into everyday settings. Encouraging participants and their families to continue practicing therapeutic activities at home and within their communities reinforces motor learning and fosters long-term functional gains [12,15,35]. Furthermore, the systematic review conducted by Lucas et al. [14] underscores the urgent need for high-quality intervention trials to refine and expand evidence-based strategies aimed at enhancing gross motor function in children and adolescents with cerebral palsy. The findings emphasize the necessity of developing and implementing interventions that are not only effective but also adaptable to diverse environments, ensuring broader accessibility and applicability in real-world settings.

The findings of this study will help to highlight the importance of family/child/adolescent-centered interventions tailored to their specific needs. Incorporating intervention sessions within the child’s or adolescent’s natural environment—whether at home or in the community—enhances the relevance and applicability of therapy, promoting greater engagement and functional improvements. This approach is expected to facilitate meaningful progress in essential motor skills, fostering independence and improving overall quality of life. Furthermore, by integrating therapy into daily routines, this model encourages sustained participation and long-term benefits beyond the structured intervention period [52].

This RCT might have some limitations. One limitation might be considering the use of muscle relaxants, apart from botulin toxin, before study participation, which could potentially influence the intervention outcomes. Future research should consider tracking these variables to better understand their impact on the rehabilitation process and ensure more comprehensive analyses. Another limitation may be that families need to find a space and time at home in order to participate in the telecare program. Besides, participation requires internet access and the ability to use the computer and also time away from work, which may not be possible for some families.

The proposed RCT will provide valuable insights into how improvements in gross motor function, gait, and balance can positively impact participation skills in children and adolescents with CP aged 6 to 17 years. By focusing on these key areas of motor development, this study aims to highlight the potential for enhanced functional independence and greater engagement in daily activities, both at home and in the community. Ultimately, the findings could contribute to the development of more effective, targeted interventions that promote improved quality of life and increased participation in social, educational, and recreational activities for individuals with CP.

## 4. Conclusions

The implementation of a multimodal PT intervention that combines face-to-face sessions of GDT and TGT and online sessions of a telecare family-centered EP, which includes AOT activities, may be effective to improve participation, gross motor function, gait and balance in children and adolescents with CP.

## Figures and Tables

**Table 1:** Overview of the assessments at each time-point.

	Baseline Assessment (A_0_)	Intermediate Assessment (A_1_)	Follow-Up Assessment (A_2_)
Descriptive characteristics	x		
Participation	x	x	x
Gross Motor Function	x	x	x
Gait speed and endurance	x	x	x
Static and dynamic balance	x	x	x
Functional Goals	x	x	x

## Data Availability

The data generated in this study will be included in the results of the published article.
